# Effects of Combination of Estradiol with Selective Progesterone Receptor Modulators (SPRMs) on Human Breast Cancer Cells *In Vitro* and *In Vivo*

**DOI:** 10.1371/journal.pone.0151182

**Published:** 2016-03-24

**Authors:** Hareesh B. Nair, Bindu Santhamma, Naveen K. Krishnegowda, Kalarikkal V. Dileep, Klaus J. Nickisch

**Affiliations:** 1 Evestra, Inc, 14508 Omicron Drive, San Antonio, TX 78245, United States of America; 2 Department of Obstetrics and Gynecology, University of Texas Health Science Center at San Antonio, 7703 Floyd Curl Drive, San Antonio, Texas 78229, United States of America; 3 School of Chemistry, Indian Institute of Science Education and Research, Thiruvananthapuram, 695016, India; UCSF / VA Medical Center, UNITED STATES

## Abstract

Use of estrogen or estrogen / progestin combination was an approved regimen for menopausal hormonal therapy (MHT). However, more recent patient-centered studies revealed an increase in the incidence of breast cancer in women receiving menopausal hormone therapy with estrogen plus progestin rather than estrogen alone. Tissue selective estrogen complex (TSEC) has been proposed to eliminate the progesterone component of MHT with supporting evidences. Based on our previous studies it is evident that SPRMs have a safer profile on endometrium in preventing unopposed estrogenicity. We hypothesized that a combination of estradiol (E2) with selective progesterone receptor modulator (SPRM) to exert a safer profile on endometrium will also reduce mammary gland proliferation and could be used to prevent breast cancer when used in MHT. In order to test our hypothesis, we compared the estradiol alone or in combination with our novel SPRMs, EC312 and EC313. The compounds were effectively controlled E2 mediated cell proliferation and induced apoptosis in T47D breast cancer cells. The observed effects were found comparable that of BZD *in vitro*. The effects of SPRMs were confirmed by receptor binding studies as well as gene and protein expression studies. Proliferation markers were found downregulated with EC312/313 treatment *in vitro* and reduced E2 induced mammary gland proliferation, evidenced as reduced ductal branching and terminal end bud growth *in vivo*. These data supporting our hypothesis that E2+EC312/EC313 blocked the estrogen action may provide basic rationale to further test the clinical efficacy of SPRMs to prevent breast cancer incidence in postmenopausal women undergoing MHT.

## Introduction

As per the scientific statement of the Endocrine Society based on the data from the various Women’s Health Initiative studies, the primary areas of benefit of MHT included relief of hot flashes and symptoms of urogenital atrophy and prevention of fractures and diabetes and, on the other hand, risks included venothrombotic episodes, stroke, and cholecystitis (1). In a WHI study, a subgroup of women starting MHT between ages 50 and 59 or less than 10 years after onset of menopause, an additional benefit of reduction of overall mortality and coronary artery disease is included. In this subgroup, estrogen plus some progestogens increased the risk of breast cancer, whereas estrogen alone did not. The statement also included that women of average age 63 is not conclusive enough to calculate risks and benefits of MHT in women starting shortly after menopause. At the present time, assessments of benefit and risk in these younger women are based on lower levels of evidence [1, 2]. The above conclusive statement from the Endocrine Society has been revisited recently by Jordan et al and provided evidences that medroxyprogesterone acetate (MPA), the progestin used in the WHI study, is a rather weak antiglucocorticoid but is effective in reducing the activation of estrogen induced apoptotic genes [3,4]. Emerging scientific evidences also suggests that PR action is context-dependent such as hormone exposure (presence versus absence of estrogen) [5, 6], organ site (proliferative in breast versus antiproliferative in uterus) [7], Ligand dependent (growth factor or kinase dependent) [8] and cross reactivity to other nuclear receptors (Onapristone- liver toxicity) [9, 10].

With these recent studies, it is difficult to interpret that the progestogen component of MHT is an important component of breast cancer risk. In this context, our approach is to utilize the classical results that derived from the WHI study to choose a progestin that has negligible levels of antiglucocorticoid activity and, with a suitable spectrum of progesterone receptor (PR) binding ability more towards PR agonism, would be a better alternative to use in combination with estrogen for MHT. The potential action of SPRMs were studied in detail previously with compounds such as Asoprisnil [11–13]. Based on the literature, we hypothesize that the ideal SPRM that could potentially be used in MHT could desirably possess the following characteristics: 1. No antiglucocorticoid receptor affinity, 2. Bind to PR and induce transcriptional activation as well as transcriptional repression in target tissue dependent manner/ desirable receptor binding profile, 3. Antiproliferative or cytostatic with regard to E2 induced cell proliferation, 4. Reduced effect on endometrium and vaginal epithelium (in terms of endometrial hyperplasia and vaginal atrophy respectively) and, 5. Prevent occult tumors in breast (breast cancer prevention).

It is documented and hypothesized in recent literature that MHT with E2 plus progestin such as MPA did not induce breast cancer development, however, promote the development of occult breast tumors that are too small to be detected on a mammogram [14]. Even though the mutagenic effect of E2 is well established in various studies [15–19], the rationale for the use of progestoegens in women with intact uterus is to oppose the estrogenic effect of uterine stimulation and to prevent uterine cancer. We hypothesize that using a suitable well profiled (as above) SPRM would inhibit the growth of such occult tumors/ breast cancer risk with E2 combination as well as expected to block occult neoplasms in postmenopausal women undergoing MHT/HRT.

Various antiprogestins with antiproliferative activity in both *in vitro* and *in vivo* has been reported, like RU 486, Onapristone and ZK 230211. [20]. However, all of these compounds belong to the class of pure progesterone receptor antagonists that lack partial agonistic activity. Such compounds are therefore not suited for a combination therapy with estrogens because this would lead to unopposed estrogenicity in the endometrium, leading to conditions such as hyperplasia or endometrial cancer [21, 22]. Patients who received up to four, 3-month long, courses of Ulipristal acetate (UPA) 10 mg daily, immediately followed by 10-day double-blind treatment with NETA (10 mg daily) or placebo, effectively controlled bleeding and fibroids shrunk in patients with symptomatic fibroids. Ulipristal is, however, approved only for 3 month treatment [23]. On the other hand, Asoprisnil developed as the first mesoprogestin, or SPRM, was given to women for 2 years without occurrences of hyperplasia [24].

A more recent approach for the treatment of menopausal symptoms is described in the US patent 6,479,535. A combination of conjugated estrogens (CE) with selective estrogen receptor modulator (SERM) like Bazedoxifene (BZD) emerged as a novel approach. This combination has been described to have positive effects on menopausal symptoms, and the ability to block the growth of occult breast cancer neoplasms in postmenopausal women which would lead to an overall reduction in tumor incidence [25, 26].

The disadvantage of the above combination might be inferior bleeding profile compared to standard estradiol progestin combinations but to prove in years to come with long term studies. In a group of postmenopausal women, the incidence of irregular bleeding during cycle 9 is 11% for a 1mg E2/0.5mg NETA combination, and 25. 8% for 0.625mg CE (conjugated estrogen)/ 5 mg MPA. The cumulative rate of amenorrhea in postmenopausal women is about 90%, and is around 80% in perimenopausal women [27]. Bazedoxifene / conjugated estrogens showed only an 83% incidence of amenorrhea in the first year of treatment in postmenopausal women [28].

Hence, there is a high medical need for an effective treatment of menopausal symptoms which offers excellent bleeding control for postmenopausal and perimenopausal women. In addition, such treatment should also provide a preventive effect on the occurrence of breast cancer [29]. In the current study, we hypothesize that our novel SPRMs, EC312/313 antagonize the effect of E2 on breast cancer growth *in vitro* and benign mammary gland proliferation *in vivo*. To address this issue, we systematically examined the effects of E2 and EC312/313 on T47D breast cancer cells and tested whether our compound blocked the effect of benign mammary gland proliferation induced by E2.

## Materials and Methods

### Compounds

17β Estradiol, purchased from Sigma Aldrich (St. Louis, MO) was dissolved in DMSO and diluted to various concentrations. Bazedoxifene, purchased from Selleckchem (Houston, TX). EC312, EC313 and EC317 were synthesized in-house at Evestra, Inc. based on structure activity relationship studies.

### Cell culture

The human breast cancer cell line, T47D (ATCC, Manassas, VA), was routinely maintained in RPMI medium with L-glutamine and 5% fetal bovine serum (FBS) (Life Technologies, Grand Island, NY) for testing premenopausal condition and 5% dextran coated charcoal stripped serum (DCC-FBS) (Life Technologies, Grand Island, NY) in the absence of phenol red was used for testing conditions includes postmenopausal scenario [23]. Cell lines such as MCF-7 and T47D form tumors in the presence of estrogen and, growth can be inhibited by anti-oestrogen therapy. We have chosen T47D cells in our studies since they express more stable levels of PR apart from ER levels.

### Cell viability studies

For determining cell number, T47D cells were plated in 6-well plates at the density of 30,000 cells per well in RPMI medium containing 5% FBS. The medium was aspirated two days later and replenished with fresh media along with treatments. Treatment from day 1 were replaced on day 3 along with fresh media. On day 6, the cell numbers were counted using TC-20 automated cell counter (Bio-Rad, Hercules, CA) [25].

### Determination of cell proliferation by 5-bromo-2’-deoxyuridine (BrdU) incorporation

T47D cells were plated in 12-well plates at a density of 80,000 cells per well. Two days later the culture medium was replaced with phenol red free RPMI medium containing 5% charcoal/dextran treated FBS for 24 hours for starvation. The cells were then treated with test compounds at the indicated concentrations for 24h [25]. Cell proliferation is based on the measurement of BrdU incorporation during DNA synthesis using Cell proliferation ELISA kit (Roche, Indianapolis, IN).

### Receptor binding studies

#### PR Transactivation assay

The transactivation assay was carried out in breast cancer (T47D) cells transiently transfected using X-tremeGENE HP DNA transfection reagent (Roche, Indianapolis, IN) with PR- reporter (Qiagen cat.no. CCS-6043L). The cells were grown either in absence (negative control) or presence of increasing concentrations of EC compounds (10pM- 500nM). To determine agonistic activity as a positive control, cells were treated with the synthetic progestin R5020. For the determination of the antagonistic activity, cells were treated with 100nM R5020 and, in addition with increasing concentrations of EC compounds (10pM- 50nM). Mifepristone (RU 486) was taken as a positive control.

The luciferase activity was determined in cell lysates and is measured as RLU (relative luminescence unit). All the data are expressed as % response, as mean value ± standard deviation (n = 3), as maximal effective concentration (EC_50_) or half-maximal inhibitory concentration (IC_50_) values, respectively for PR agonism and antagonism [30].

#### GR transactivation assay

Antiglucocorticoid activity was determined using *select screen* assay system (Invitrogen-Life Technologies) as described in our previously published studies [21, 22, 31].

### Determination of apoptosis by cell death by Caspase-Glo assay

Caspase-3/7 activity was measured using Caspase-Glo assay kit (Promega), as described before [32]. Briefly, the cells were treated with test compounds and homogenized in homogenization buffer (25 mmol/L HEPES, pH 7.5, 5 mmol/L MgCl_2_, and 1 mmol/L EGTA), protease inhibitors (Sigma, and the homogenate was centrifuged at 13,000 rpm at 4°C for 15 minutes. To 10 μL of the supernatant containing protein was added to an equal volume of the assay reagent and incubated at room temperature for 2 hours. The luminescence was measured using Fluorskan-FL fluorescence/ luminescence plate reader (Thermo-Fisher scientific, Austin, TX).

### Determination of gene expression by quantitative real-time PCR

T47D cells grown in 60-mm dishes were cultured in phenol red-free RPMI medium with 5% DCC-FBS for 24 hours and treated with BZD, E2 and EC compounds for 24hours before RNA extraction. Total RNA was extracted and purified using the Qiagen RNeasy plus (Valencia, CA, USA) with a genomic DNA removal step as per manufacturer’s protocol. Reverse transcription (RT) was carried out using the Applied Biosystems kit (Foster City, CA, USA). Real-time PCR and subsequent analyses were carried out using Smartmix PCR beads (Cepheid, Sunnyvale, CA, USA) with SybrGreen in the Cepheid SmartCycler to detect testing genes and the housekeeping gene (GAPDH) transcripts, as described previously [33]. The primer sets used for the study were listed as supporting information (S1 Primers). PCR reactions using testing genes and GAPDH primer sets gave unique melt peaks, indicative of discrete amplification products. Real-time PCR assays were performed in duplicates and repeated at least three times.

### Determination of protein expression by Western blot analysis

Expression of various proteins was carried out using Western blot analysis. Total protein was isolated from the cells in RIPA buffer (Sigma-Aldrich, St Louis, MO). Equal amount of protein (60 μg) from representative samples were separated on a denaturing polyacrylamide gel and transferred to a nylon membrane. Nonspecific binding of antibodies was blocked by incubation (overnight, 4°C) in TBS containing 0.1% Triton X-100 (TBST) and 5% nonfat dry milk. Membranes were then incubated with respective primary antibodies in TBST and 5% milk overnight at 4°C. Specific binding to target protein was visualized by using species-specific IgG followed by chemiluminescent detection and exposure to enhanced chemiluminescence hyperfilm (Amersham, Piscataway, NJ). Antibodies for Western analysis were purchased from NeoMarkers-Cyclin D1, ERα (Fremont, CA), Santa Cruz Biotechnology- Actin (Santa Cruz, CA) and Cell signaling Technology- MAPK, p-MAPK, AKT, pAKT (Lane Danvers, MA) [34].

### Animal experiment

United States Department of Agriculture and Association for the Assessment and Accreditation of Laboratory Animal Care guidelines were followed, and the studies were approved by the Institutional Animal Care and Use Committee of University of Texas Health Science Center at San Antonio. Four-week-old, C57BL/6 female mice, obtained from Charles River Laboratories (Germantown, MD), were ovariectomized and administered E2, EC313 alone and in combination. All standard protocols to alleviate suffering, including methods of anesthesia and euthanasia was maintained during the experiment. The animals were randomly grouped in to 4 groups as vehicle control, E2, E2+0.1 and 1mg/kg EC313. Each group was comprising 6 animals. They were sacrificed 4 weeks later to assess mammary gland development, uterine weight and gene expression. E2 was given by daily injection (sc) at the dose of 5 μg/kg in dimethyl sulfoxide/PBS (1:1) solution. EC313 at doses of 0.1 and 1.0 mg/kg were administered intraperitoneally (i.p). The doses were selected based on our *in vitro* experiments and from published literature [35].

Animals were fed standard mouse chow with water *ad libitum*. During the experiment, body weights were assessed weekly. At the termination of the experiment, uteri were excised, trimmed of adherent fat and weighed. Representative single inguinal mammary glands were excised, fixed in Carnoy’s solution as described below and stained for morphological analysis. The other inguinal mammary gland from the same animals was snap frozen in liquid nitrogen and stored at -80°C for gene expression studies.

### Whole mount analysis

Whole mounts of mammary glands were prepared essentially as described previously [36]. The left fourth abdominal mammary glands were dissected at necropsy and spread onto glass slides, dried briefly, fixed in Carnoy's fixative for at least 30 min, and stored in 70% ethanol. Glands were then stained with carmine alum, dehydrated, and coverslipped. The length of time for staining whole mounts with carmine alum was determined empirically to achieve maximum contrast between the ductal epithelium and the surrounding adipose tissue.

### Statistics

All quantitative data are expressed as the mean ±SD. Statistical significance was determined by Student’s *t* test.

## Results

### Effect of EC312and EC313 on total cell number

**Fig 1:**
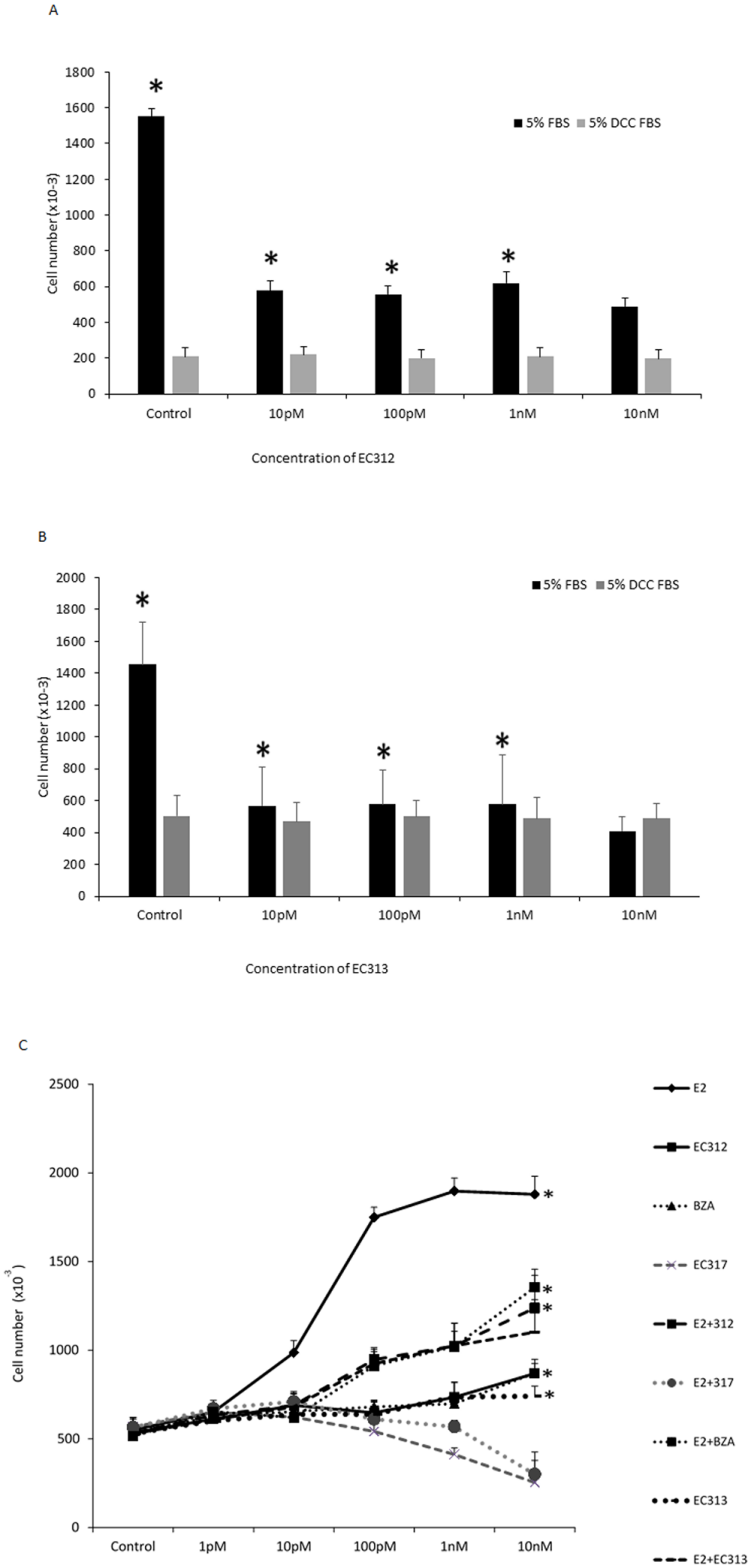
Antiestrogenic effect of EC312/313 on cell viability and proliferation in vitro. A-B) Antiestrogenic effect of EC312 and EC313 on cell growth in the presence and absence of endogenous estrogens. T47D cells were plated at 30,000 cells per well in 5% FBS-RPMI and grown for 2 days before treatment. The cells were then incubated with EC312 and EC313 at indicated concentrations for 5 days and counted for cell number. *P<0.01 vs control. **C)** The compounds were treated as per the conditions above with and without E2 (0.1nM). Tested compounds were found to inhibit E2 induced cell proliferation as comparable to that of BZD (10nM). EC317 was used as a pure PR antagonist (*P<0.01 vs control).

Since antiprogestins and antiestrogens demonstrated to have deferential effects on cultured breast cancer cells in the presence and absence of estrogenic compounds in the serum and in the medium such as phenol red [29], we have evaluated whether our compounds elicit estrogen agonistic/antagonistic actions in the presence and absence of endogenous estrogen. Initially we have analyzed the presence of endogenous estrogens (RPMI with phenol red and 5% FBS) and found that each concentration of EC312 and EC313 reduced cell number by approximately 60% with the maximum effect observed at 10nM. On the other hand, in estrogen depleted medium (phenol red-free RPMI with 5% DCC-FBS), neither compounds showed estrogen agonistic or antagonistic properties (Fig 1A and 1B). Further we have evaluated whether EC compounds have inhibitory effect on peak stimulated dose of E2 in breast cancer cells. We have adopted this concentration of E2 as 0.1nM based on our E2 dose dependent study in T47D cells as well as published elsewhere (25). We have used different concentrations (1pM, 10pM, 100pM, 1nM and 10nM) of EC312, 313, 317, RU486 and BZD. EC312 and EC313 were used as SPRMs, whereas EC317 served as potent pure antiprogestin without glucocorticoid activity [21]. BZD is a SERM used currently in the clinics for MHT in combination with conjugated equine estrogens (CE) [37]. EC312 and EC313 were found to inhibit E2 induced cell growth as comparable to that of BZD (Fig 1C). The complete abrogation of cell proliferation was noticed in EC317 treated cells as expected.

### Effect of EC312and EC313 on cell proliferation

**Fig 2:**
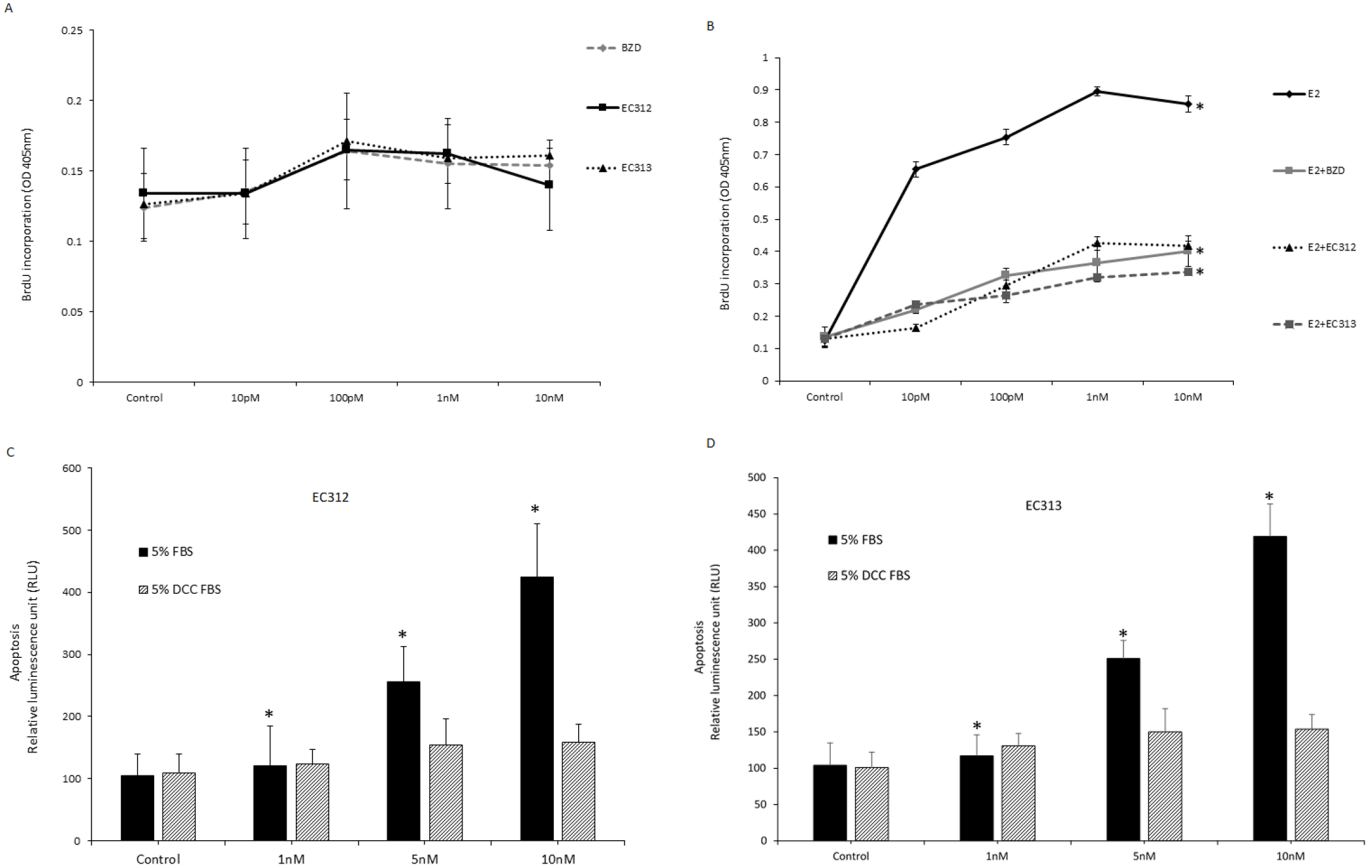
EC312 and EC313 inhibiting DNA synthesis. A-B) Effect of EC312 and EC313 on cell proliferation as assessed by BrdU incorporation. T47D cells were plated in 96-well plates at a density of 10,000 cells per well. Two days later the culture medium (5% FCS-RPMI) was replaced with phenol red-free RPMI with 5% DCC-FCS. After 24 hours of starvation, the cells were treated with increasing doses of the compounds alone or in combination with E2 (0.1nM). The results are expressed as average OD value of quadruplicate wells (*P<0.01 vs control). C-D) Effect of EC312 and EC313 in inducing apoptosis in the presence or absence of endogenous estrogen in T47D cells. T47D cells were plated in 12 well plated at a density of 80,000 cells/well. Two days later culture media were replaced with phenol red-free RPMI containing 5% FBS or 5% DCC-FBS. After 24 hours of starvation, cells were treated with test compounds at increasing concentrations for 48 hours. Apoptosis was assessed by Caspase-Glo assay kit (Promega). The results were expressed as OD±SEM of 2 wells per treatment (*P<0.01 vs control).

In order to assess whether the observed the inhibition of cell viability with EC312 and EC313 treatment is due to decrease in cell proliferation by inhibiting DNA synthesis, we have employed BrdU incorporation assay. Like BZD, EC312 and EC313 didn’t exhibited any estrogen agonistic properties in the absence of estrogen (DCC-FBS +phenol red-free medium) (Fig 2A), whereas E2 (0.1nM) induced BrdU incorporation was blocked by EC compounds (Fig 2B). This result clearly shows that the above EC compounds do not possess E2 agonistic effect by itself, but inhibited E2 induced cell proliferation by inhibiting DNA synthesis.

### Selectivity of EC312 and EC313 for steroid receptors

In order to determine *in vitro* glucocorticoid and progestational activity of EC312 and EC313, transactivation studies were performed. RU486 served as standard substance for the antiglucocorticoid and antiprogestational activity. Data are reported as IC 50 and are calculated from two set of independent experiments. All tested compounds showed a good dissociation between antiprogestational and antiglucocorticoidal activity. EC312 and EC313 showed clear signs of agonistic activity in *transactivation* assays (Table 1).

**Table 1:** Glucocorticoid receptor (GR) antagonistic activity, Progesterone receptor (PR)—agonistic and antagonistic activity is evaluated and IC50 values as well as agonistic/antagonistic ratios were noted. ND- Not determined.

Compounds tested	IC50 GR Agonism (Control-Dexamethasone) (nM)	EC50 PR Agonist (Control- R5020) (nM)	IC50 PR Antagonism (Control-RU486) (nM)	Ratio EC50/IC50 PR Agonistic/Antagonistic mode
Mifepristone (RU486)	0.969	>100	1.14	>88
Ulipristal Acetate (CDB2914)	ND	21.5	0.65	33.1
21,21-difluoro-11β-[4’-(3’-pyridyl)phenyl]-17,23-epoxy-19,24-dinor-17α-chola-4,9,20-triene (EC312)	3.08	51.5	0.625	82.4
21,21-difluoro-11β-[4’-(3’-furanyl)phenyl]-17,23-epoxy-19,24-dinor-17α-chola-4,9,20-triene (EC313)	12.8	4.5	0.5	9
11β-(4’-(1-imidazolyl)phenyl)-17β-hydroxy-17-(1,1,2,2,2-pentafluoroethyl)-estra-4,9-diene-3-one (EC317)	4.11	>100	0.47	>217

These *in vitro* data correlate with *in vivo* data in guinea pigs that characterize EC-317, as a very pure antagonist and EC313 as mesoprogestin with strong partial agonistic activity [31]. In the case EC312, the receptor binding studies does not correlate with our previously published *in vivo* studies in guinea pigs [38]. This might be due to the difference in binding preference as seen in *in silico* studies (S1 Text, S1 Fig, S1 Table) or preference in the recruitment of co-factor/co-repressor and exerting non-genomic action. However, to avoid this disparity we have focused our *in vivo* studies in EC313.

### Effect of EC312 and EC313 in inducing apoptosis in breast cancer cells in the presence and absence of endogenous estrogen

**Fig 3:**
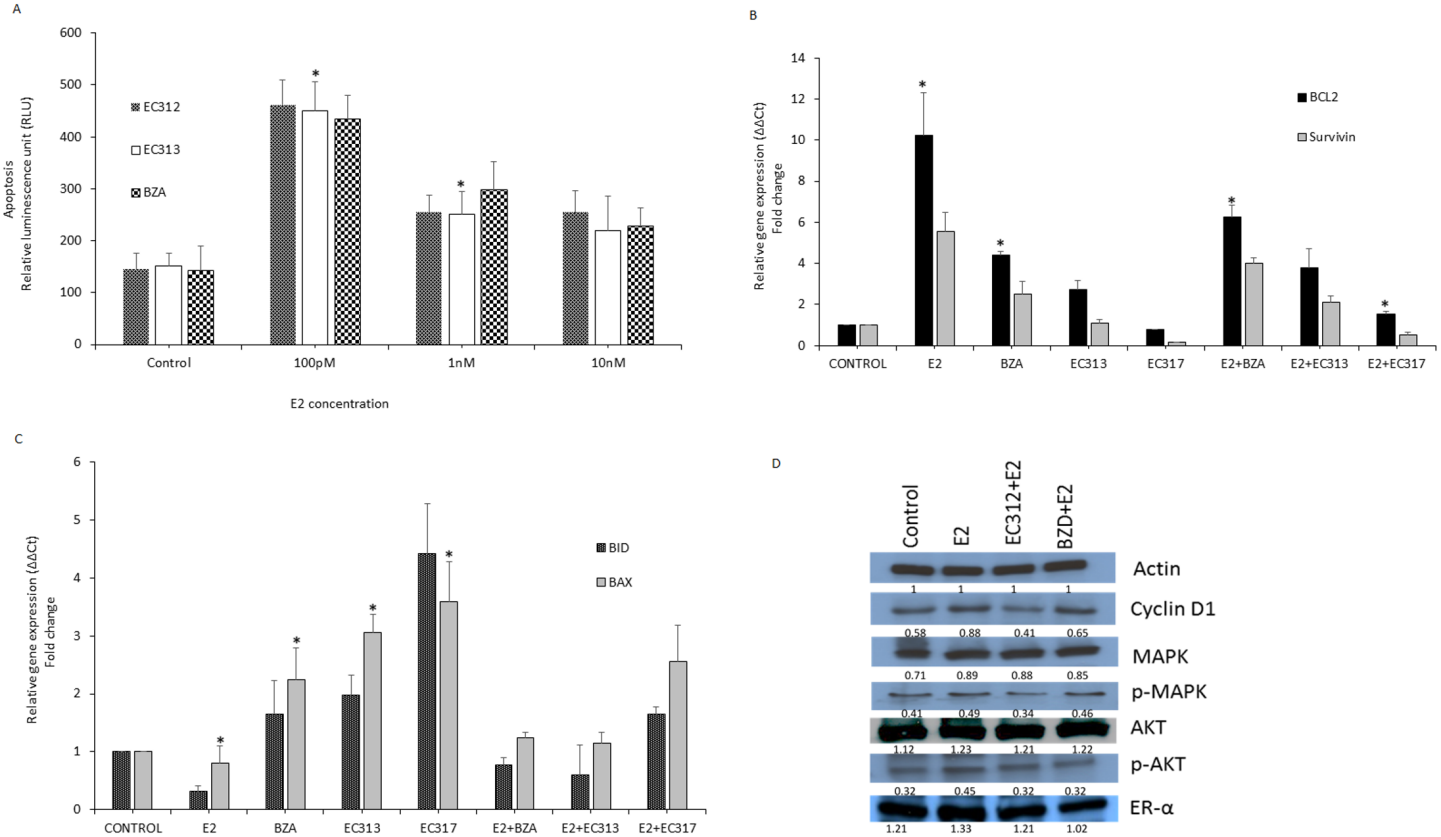
Effect of EC312 and EC313 on apoptosis, gene signatures of proliferation and protein expression levels. A) Effect of EC312 and EC313 (10nM) on apoptosis in the absence of endogenous estrogen and increasing concentrations of exogenous E2. We have selected the above concentration based on the fact that EC312 and EC313 abrogated E2 induced cell proliferation as comparable to that of BZD at 10nM). T47D cells were plated in 12 well plated at a density of 80,000 cells/well. Two days later culture media were replaced with phenol red-free RPMI containing 5% FBS or 5% DCC-FBS. After 24 hours of starvation, cells were treated with test compounds at increasing concentrations for 48 hours. Apoptosis was assessed by Caspase-Glo assay kit (Promega). The results were expressed as OD±SEM of 2 wells per treatment (*P<0.01 vs control). B) Effect of EC313 on gene signatures of proliferation. Gene expression of T47D cells in response to EC313 for anti-apoptotic genes. T47D cells were grown in phenol red-free RPMI medium containing 5% DCC-FBS for 24 hours and then treated with indicated doses of test compounds alone or in combination with E2 (0.1nM) (*P<0.01 vs E2-control). C) Effect of EC313 on gene signatures of proliferation. Gene expression of T47D cells in response to EC313 for pro-apoptotic genes. T47D cells were grown in phenol red-free RPMI medium containing 5% DCC-FBS for 24 hours and then treated with indicated doses of test compounds alone or in combination with E2 (0.1nM) (*P<0.01 vs E2-control). D) Effect of EC312 and BZD on the expression of ER, Cyclin D1 and phosphorylation of MAPK and AKT. T47D cells grown in 60-mm culture dishes in phenol red-free RPMI medium containing 5%DCC-FBS and treated with E2 (1nM) alone or in combination with EC312 or BZD (100nM) for 24 hours before preparation of cell lysate and analysis by western blot and quantification normalized by beta-actin.

We have examined whether EC312 and EC313 induce apoptosis in the presence or absence of endogenous estrogen in T47D cells. Both compounds dose dependently induced apoptosis in the presence of endogenous estrogen, however the compounds had little effect on inducing apoptosis under estrogen depleted conditions (Fig 2C and 2D). We have also examined 10nM concentration of the compounds with increasing concentrations of E2. Each higher concentration of E2 reduced apoptosis and effect was comparable to that of BZD, a known SERM (Fig 3A).

### EC312 and EC313 altered gene signatures of proliferation

To confirm the above seen effect of the compounds in apoptosis, we have analyzed the pro-apoptotic and anti-apoptotic genes in response to treatment with EC compounds in T47D cells. Anti-apoptotic genes such as BLC2 and survivin were found to be down regulated with the treatment. EC313 treatment with E2 reduced the expression of BCL2 and survivin. The observed effect was abrogated by EC317, a pure PR antagonist [31]. On the other hand proapoptotic genes such as BAX and BID expression levels were upregulated with EC313 treatment and a maximum induction of apoptosis was observed with the EC317 treatment. The effect was reduced by 0.5 fold with BZD in the absence of E2, however, when combined with E2, EC compounds elicit comparable results with BZD (Fig 3B and 3C). These results provide potential mechanism to explain why EC compounds blocked anti-apoptotic effects of E2. We have observed the similar results with EC312.

### Mechanism of action of EC312/EC313 at protein level

To determine the observed inhibitory action of the T47D cell proliferation by EC312/313 we have checked proliferation markers such as cyclin D1 and non-genomic effectors such as MAPK and AKT phosphorylation status. Cells treated with E2+ EC312 down regulated cyclin D1 levels when compared to E2 treated cells. The observed effect of EC312 was superior when compared to BZD (Fig 3D). The ER levels were not changed as expected with the EC312 treatment and found reduced as that of BZD treated group. The observed effect might be due to activity of EC312 by blocking E2 induced- ER regulated genomic effects on breast cancer cell proliferation. Apart from the genomic effects, EC312 treatment reduced the E2 induced AKT activity as non-genomic effect of E2. The levels of phosphorylated AKT was reduced in EC312 treated T47D cells. Since it has been shown that E2 induced AKT phosphorylation is mediated through MAPK [39], we have tested phosphorylation of MAPK activity in these cells. Phosphorylated MAPK levels were found down regulated when compared to unchanged total MAPK levels in EC312 treated cells along with E2 (Fig 3D). From these results is clear that EC312 and did not enhance E2 induced breast cancer cell proliferation, but reduced downstream effectors of E2 induced mitogenic effects. The similar results with EC313 will be published along with T47D tumor xenograft study.

### EC313 reduced E2 induced uterine weight mammary gland ductal branching in mice

**Fig 4:**
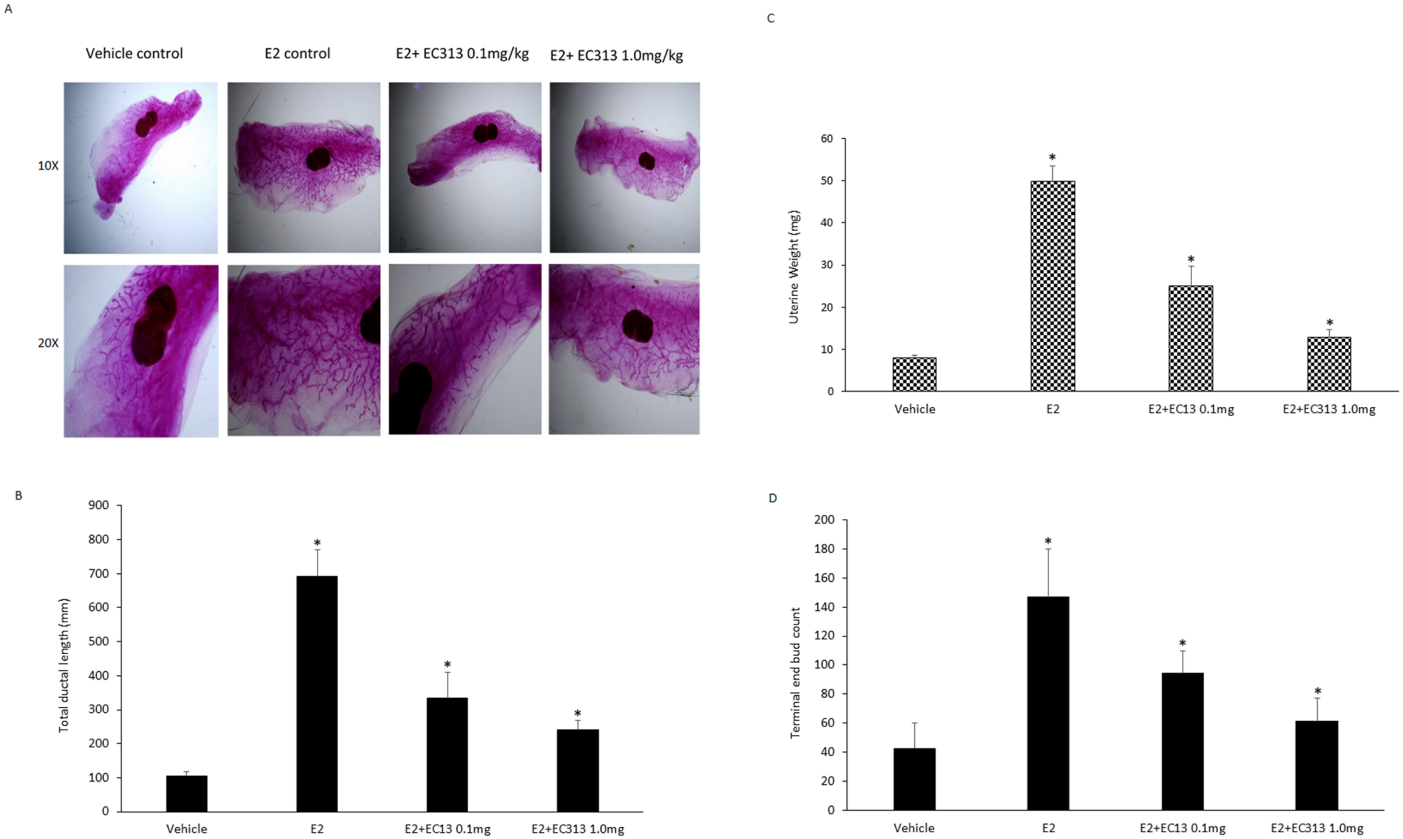
Effect of EC13 on mammary gland morphology and uterine weight. A) Whole mounts of mammary glands of overiectomized C57BL/6 mice treated with E2, EC313 0.1 and 1.0 mg/kg. Magnification- upper panel-10X, lower panel-20X. B) Uterine weight of mice treated for 4 weeks with E2, EC313 0.1 and 1.0 mg/kg. Data represented are average uterine weight in milligrams ±SD (*P<0.001 vs E2-control). C-D) Total duct length and terminal end bud counts in the mammary gland whole mounts after 4 weeks of the treatment with test compounds (*P<0.001 vs E2-control).

In order to determine the *in vivo* estrogenic action of EC313 in uterine weight and benign breast development, we have used standard uterine weight bioassay [40, 41] and mammary gland whole mount analysis in ovariectomized mice. Five different measurements were obtained from each whole-mount image. Ductal length (millimeters) was measured by drawing and measuring a straight line caliper from the most distal point of the ductal network to the nipple and end buds were identified as large bulbous profiles located at the termini of ducts. EC313 dose dependently reduced the ductal branching and terminal end buds in whole mount analysis. E2 stimulated uterine weight was reduced by two fold when combined with EC313 0.1mg/kg and three fold with 1.0mg/kg dose of EC313 (Fig 4A, 4B, 4C and 4D).

**Fig 5:**
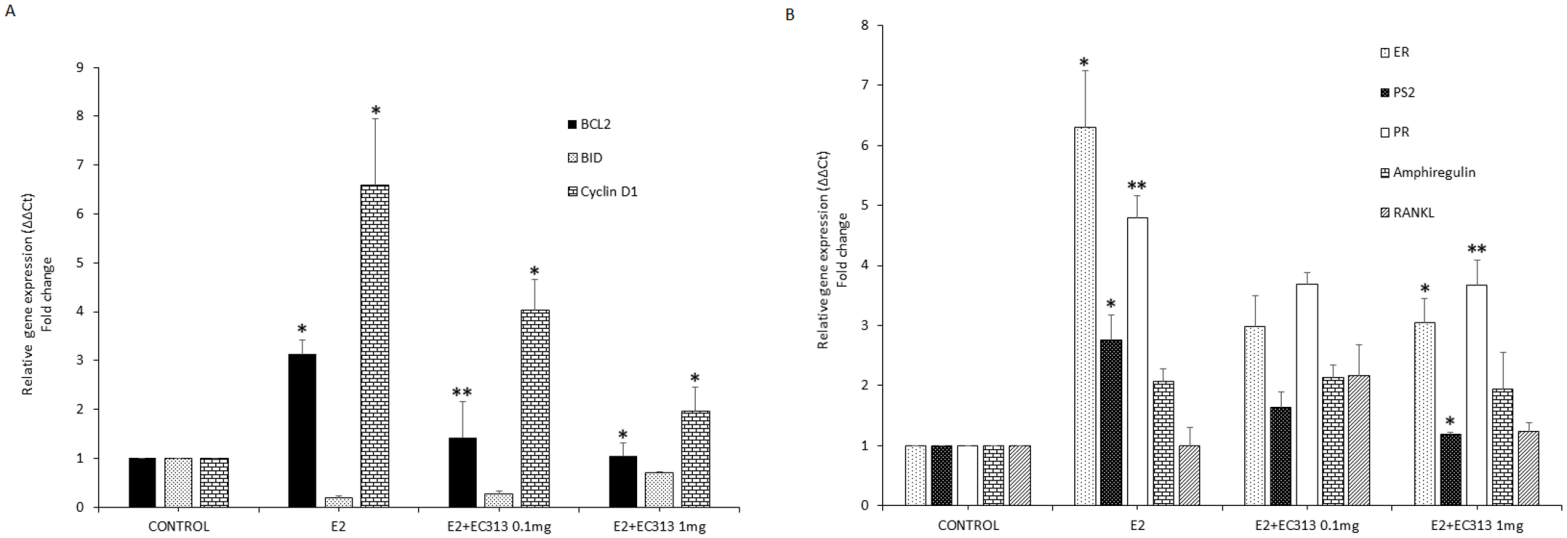
Effect of EC313 on gene expression of proliferation, apoptotic markers and PR regulated genes. A-B), Gene expression in mammary glands of overiectomized C57BL/6 mice treated with E2, EC313 0.1 and 1.0 mg/kg. Columns are average of relative amount (ΔΔCt) of tested mRNA ± SD (n = 6) (**P<0.01 vs E2-control, *P<0.001 vs E2-control).

The reduced estrogenic effect by the treatment of EC313 was further confirmed by the gene expression studies. The mechanism of EC13 induced apoptosis was confirmed as inhibition of proliferation marker-cyclin D1, anti-apoptotic genes such as BCL2 and pro-apoptotic gene, BID as seen before in *in vitro* T47D cell model (Fig 5A). The E2 regulated genes such as cyclin D1 and PS2 were upregulated in the mammary glands of E2 treated animals, whereas those were downregulated with EC313 treatment combined with E2. Since our compounds is an SPRM, the classical PR regulated genes such as amphiregulin (AREG) and RANKL were upregulated in a moderate fashion with EC313 treatment at lower doses but significantly inhibited at higher dose (Fig 5B).

## Discussion

After the discovery of reduced breast tumor incidence after ovariectomy by British Surgeon, George Beatson [42], pharmacological inhibitors of estrogen and progesterone were developed. PR antagonists showed promising results in second line and first line breast cancer treatment, but the development was stopped side effects such as liver toxicity, on the other hand compound that inhibited estrogen signaling such as tamoxifen in 1970s and later 20 years after aromatase inhibitors became mainstay of breast cancer therapy. This previous knowledge has been utilized to develop contraceptives and menopausal hormonal therapy (MHT) in pre and post-menopausal women respectively using agonist of estrogen and progesterone. Addition of progestogen to the estrogen component of MHT has resulted an increased risk of breast cancer in postmenopausal women in Women’s Health Initiative (WHI) study [2, 43]. Strategies to eliminate the progestogen component in MHT was carried out lately using selective estrogen receptor modulators (SERMS) such as BZD combined with conjugated equine estrogens (CE). This approach has been approved recently in clinics and elicit mixed results. Results from these clinical studies suggest that BZD/CE regimen is safer in terms of relieving vasomotor symptoms, reducing the risk of osteoporosis and breast cancer in postmenopausal women [44, 45]. However, studies also revealed that BZD/CE and CE/NETA has showed incidence of irregular bleeding in postmenopausal women [27, 28]. We believe that there is still an unmet need for a safer menopausal therapy that may offer a favorable bleeding and tolerability profile.

All previously synthesized progestogens were created for basic the purpose of contraception and those behaved differently due to multitude of regimens and plethora of approaches that they used. Hence one would argue that a better differentiated analysis would identify the risk factors, such as women’s age and reproductive status etc. The observed breast cancer risk in WHI study could be related to the increase in cell proliferation in the breast epithelium elicited by PR signaling. In this study we demonstrate that novel SPRM such as EC313 that decreases cell proliferation and inhibited E2 induced mitogenic effects would be a better therapeutic combination with E2 for MHT in postmenopausal women. The presented data, taken together, suggests that further studies including clinical trial to confirm our hypothesis in postmenopausal women is warranted.

Our in vivo study revealed that EC313 blocked the estrogenicity of E2 on uterus and mammary gland proliferation including ductal length, terminal end bud development and induced apoptosis. This results were matched with our previous findings classified EC313 as a mesoprogestin [38]. The context dependent progesterone action might help to explain the observed effects of EC313 on T47D breast cancer cells and benign mouse mammary glands. According this concept, target gene selectivity is not only achieved through differential recruitment to PR, but also through related co-activators and co-repressors to PR [46]. In the present study we have observed RANKL gene expression in the mammary glands of EC313 treated mice than that of E2 control animals. The expression of RANKL was dose dependently reduced with higher dose of EC313 (1mg/kg). It is demonstrated previously that PR induced RANKL expression requires STAT5A [47]. Emerging evidences also suggests that SPRMS that target RANKL pathway could inhibit breast cancer cell proliferation [48]. It is also important to note from mouse mammary gland studies that estrogen receptor-alpha (ERα) signaling controls pubertal gland development whereas PR signaling is major proliferative stimulus in adult mammary gland [7].

It has been understood from the WHI study that women taking HRT/MHT have little or no increase in breast cancer risk when taking estrogen alone and even offer a protective effect in this group have been noticed [43]. Also there is major concern of localized and widespread endometrial cancer with the use of conjugated estrogens [49]. In a recent clinical study the percentage of subjects treated daily oral dose of BZD 20mg + CE 0.45mg resulted at 0.83% of vascular disorders such as deep vein thrombosis when compared to placebo and CE+MPA group (no incidents reported) comprising a total of 6.09% serious adverse effects verses 3.9% for placebo and CE+MPA group (clinicaltrials.gov/ct2/show/results/NCT00242710). We believe that choosing right combination of progesterone receptor modulator (SPRM) in terms of their differential receptor binding capabilities would be a better modality to adopt in MHT as an alternative approach. In summary it can be concluded that there is still a high medical need for an effective treatment of menopausal complaints with an excellent bleeding control for postmenopausal and perimenopausal women. In addition such treatment should have a preventive effect on the occurrence of breast cancer. Upon considering the limitation of the present study in concluding the potential of SPRMs and estradiol (E2) combinations for MHT, we are currently conducting pharmacokinetic and toxicity studies of our lead compounds to ensure the endocrinological and general safety of such combination *in vivo*. Warranting further studies in breast cancer prevention models, EC313 could potentially use in MHT to antagonize anti-apoptotic action of estrogen, preventing occult breast tumors, thereby reducing the future risk for breast cancer and preserving the safer bleeding profile in postmenopausal women.

## Supporting Information

S1 Fig Binding modes of EC312 (A) and EC313 (B) with Progesterone Receptor. (TIF)

S1 Primers (DOC)

S1 Table Binding free energies of selected ligands towards different hormone receptors. (DOCX)

S1 Text Molecular Modeling studies. (DOCX)

## Abbreviations

AREG: amphiregulin
BrdU: 5-bromo-2’-deoxyuridine
BZD: bazedoxifene
CE: conjugated estrogen
SPRM: selective progesterone receptor modulator
SERM: selective estrogen receptor modulator
ER: estrogen receptor
PR: progesterone receptor
FBS: fetal bovine serum
MHT: menopausal hormonal therapy
HRT: hormone replacement therapy
E2: estrogen
