# Supplementary figures and images for: Effects of Combination of Estradiol with Selective Progesterone Receptor Modulators (SPRMs) on Human Breast Cancer Cells In Vitro and In Vivo
Source: PLoS One. 2016 Mar 24;11(3):e0151182. doi: 10.1371/journal.pone.0151182 (PMC4806908; doi:10.1371/journal.pone.0151182)

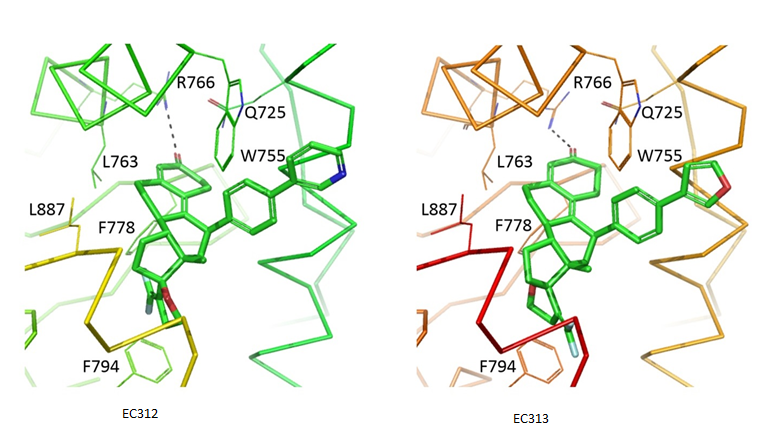

Supplement: S1 Fig — (TIF) [file pone.0151182.s001.tif]
