# Supplementary material for: Effects of Combination of Estradiol with Selective Progesterone Receptor Modulators (SPRMs) on Human Breast Cancer Cells In Vitro and In Vivo
Source: PLoS One. 2016 Mar 24;11(3):e0151182. doi: 10.1371/journal.pone.0151182 (PMC4806908; doi:10.1371/journal.pone.0151182)
Supplement: S1 Primers — (DOC) [file pone.0151182.s002.doc]

For Human samples

BCL2

5′-TGACTTCAGCTGCCTCACTT-3’ and 5′-GTTCCCACAGCTGCTCTGTA-3’

Survivin

5′-TGG CAG CTG TAC CTC AAG AA-3′ and 5′-AGC TGC TCA ATT GAC TGA CG-3′.

BID

5’-AGACGAGCTGCAGACAGATG-3’ and 5’-GGTCCATCTCATCGCCTATT-3’

BAX

5’-TAGCAAACTGGTGCTCAAGG-3’ and 5’-TCTTGGATCCAGACAAGCAG-3’

GAPDH

5′-ATG GTG AAG GTC GGT GTG AAC G-3′ and 5′-GTT GTC ATG GAT GAC CTT GGC C-3′

For Mouse samples

BCL2

5′-TATAAGCTGTCACAGAGGG-3’ and 5′-CTCTCCACACACATGACC-3’

BID

5′-TGGACTGTGAGGTCAACAACG-3’ and 5′-GCGTCCATCCCATTTCTGG-3’

Cyclin D1

5′-CACACGGACTACAGGGGAGT-3′ and 5′-CACAGGAGCTGGTGTTCCAT-3′

ERα

5’-GACCAGATGGTCAGTGCCTT-3’ and 5’-ACTCGAGAAGGTGGACCTGA-3’

PR

5’-GGTGGGCCTTCCTAACGAG-3’ and 5’-GACCACATCAGGCTCAATGCT-3’

PS2

5′-AGGAGCTGGAGGAGGAGGAG-3′ and 5′-ATGTAGAGCTGGTGGGAGGC-3′

AREG

5'-CACAGCGAGGATGACAAGGA-3‘ and 5'-GAGGATGATGGCAGAGACAAAGA-3’

RANKL

5′-TGTACTTTCGAGCGCAGATG-3′ and 5′-AGGCTTGTTTCATCCTCCTG-3′

GAPDH

5′-GAGTCCACTGGCGTCTTC-3’ and 5′-GGGGTGCTAAGCAGTTGGT-3′
