# Supplementary material for: Effects of Combination of Estradiol with Selective Progesterone Receptor Modulators (SPRMs) on Human Breast Cancer Cells In Vitro and In Vivo
Source: PLoS One. 2016 Mar 24;11(3):e0151182. doi: 10.1371/journal.pone.0151182 (PMC4806908; doi:10.1371/journal.pone.0151182)
Supplement: S1 Table — (DOCX) [file pone.0151182.s003.docx]

| Sl No. | Name of the ligands | PR | ER agonist bound form | ER antagonist bound form | GR agonist bound form | GR antagonist bound form |
| --- | --- | --- | --- | --- | --- | --- |
|  |  | Binding energies in kcal/mol | | | | |
| 1 | EC312 | -97.89 | -9.45 | -10.11 | -17.69 | -23.67 |
| 2 | EC313 | -98.43 | -8.67 | -10.07 | -17.01 | -22.18 |
| 3 | OHT | nd | -93.26 | nd | nd | nd |
| 4 | DES | nd | nd | -99.17 | nd | nd |
| 5 | DEX | nd | nd | nd | -140.37 | nd |
| 6 | RU486 | nd | nd | nd | nd | -131.32 |
| 7 | ASO | -101.60 | nd | nd | nd | nd |

*nd – docking studies has not performed

S1 Table: Binding free energies of selected ligands towards different hormone receptors
