# Supplementary material for: Effects of Combination of Estradiol with Selective Progesterone Receptor Modulators (SPRMs) on Human Breast Cancer Cells In Vitro and In Vivo
Source: PLoS One. 2016 Mar 24;11(3):e0151182. doi: 10.1371/journal.pone.0151182 (PMC4806908; doi:10.1371/journal.pone.0151182)
Supplement: S1 Text — (DOCX) [file pone.0151182.s004.docx]

In silico computational docking studies

In order to evaluate whether EC312 and EC313 are selective towards progesterone receptor (PR), a series of molecular docking investigations has been carried out. EC312 and EC313 were docked to the agonist and antagonist bound form of estrogen receptor (ER) and glucocorticoid receptor (GR). Crystal structures of human PR in complex with Asoprisnil [(ASO) (PDB ID: 2OVH)], alpha form of ER in complex with a 4-hydroxytamoxifen [(which is an agonist) (OHT) (PDB ID: 3ERT)] and diethylstilbestrol [(which is an antagonist) (DES) (PDB ID: 3ERD)], human GR in complex with dexamethasone [(which is an agonist) (DEX) (PDB ID: 1P93)] and RU-486 [(which is an antagonist) (PDB ID: 1NHZ)] were selected as receptors for the studies. These receptors were prepared using protein preparation wizard of Schrodinger suite. As a part of protein preparation, the crystallographic water molecules were deleted, hydrogen were added and minimized using OPLS 2005 force field. Similarly the ligands such as EC312, EC313, ASO, OHT, DES, DEX and RU-486 were prepared using Ligprep module. Binding affinity of all these ligands towards their parent structures were investigated using extra precision docking method followed by binding free energy calculations. Before docking studies, the ligand binding area was defined using a grid by selecting 10 Å space away from the crystallographic ligand. After docking, the best 10 poses were selected based on the G score and are used for the binding free energy calculations. Prime module of Schrodinger was used for the binding free energy calculations.

EC312/EC313 binds to PR but not to GR and ER

The molecular docking studies suggested that EC312 and EC313 are well fit in to the ligand binding domain of PR. The binding mode of these ligands is exactly similar to that of ASO (Figure 1). Even though these compounds sharing similar binding modes, EC313 exhibit slightly increased binding affinity towards PR compared to EC312. This correlates the *in vitro* results of the higher agonism of EC313 towards PR. In EC312 and EC313, the keto group located in the ‘A’ ring of the steroid scaffold is acting as an anchor to hold the whole ligand in to the ligand binding domain through a salt bridge with R766 as shown in the figure 1. Apart from this, a series of hydrophobic interactions were also observed between the ligands and proteins residues. At the same time the docking results of EC312 and EC313 towards other hormone receptors suggested that these compounds are poor binders of ER and GR. The binding mode of EC312 and EC313 in the active site of both ER and GR is in such a manner that the half portion of the ligand is buried inside the ligand binding cavity and the rest portions are exposed to the solvents. As a result, only negligible contacts are maintained with the protein residues and for the same reason the binding affinity is found to be drastically less for EC312 and EC313. The binding energies of selected agonists and antagonists towards their respective structures are found to be high (S1 Table). The close analysis of the ligand binding domain of agonist and antagonist bound form of ER and GR lead to a conclusion that there is inadequate room in the active sites to accommodate EC312 and EC313. The volumes of the selected ligands were predicted and compared with that of EC312 and 313 and found to be small. Hence a stable interaction can’t maintain for EC312 and EC313 in the ligand binding domain.
